# Impact of COVID-19 pandemic on patient satisfaction and surgical outcomes: A retrospective and cross sectional study

**DOI:** 10.1016/j.amsu.2020.08.020

**Published:** 2020-08-21

**Authors:** Thamer A. Bin Traiki, Sulaiman A. AlShammari, Mohammed N. AlAli, Nadia A. Aljomah, Noura S. Alhassan, Khayal A. Alkhayal, Omar A. Al-Obeed, Ahmad M. Zubaidi

**Affiliations:** Department of Surgery, College of Medicine, King Khalid University Hospital, King Saud University, Riyadh, 11461, Saudi Arabia

**Keywords:** COVID-19, Patient's satisfaction, Saudi Arabia, Surgeries

## Abstract

**Objective:**

The objective of this study was to evaluate the impact of the COVID-19 pandemic on patient satisfaction and surgical outcomes at King Khalid University Hospital in Saudi Arabia.

**Background:**

The COVID-19 pandemic has greatly affected health care systems across developing and developed countries. Therefore, it is important to understand its impact on various parameters of patient care as regards revised infrastructure and policies in hospitals during the pandemic.

**Method:**

It is a retrospective cross-sectional study was conducted from 13-3–2020 to 26-4-2020 at King Khalid University Hospital in Saudi Arabia. Patient satisfaction and surgical outcomes were the main outcome measures.

**Results:**

331 participants were included in the study (median age: 53 years; 70% female), and 223 completed the patient's satisfaction survey. 260 of the surgeries were non-oncolog cases (78.6%) compared to 71 oncology cases (21.4%). With respect to the surgical outcomes, 12% of the patients required admission to the ICU, and 10.9% developed postoperative complications, most of which were infectious complications. Only 1.8% (6 patients) were re-admitted to the hospital. Three patients died within 30 days post-op (0.9%), all had emergency surgery. Regarding patient satisfaction, 77.6% and 93% of the patients reported that nurses and doctors, respectively, treated them with courtesy and respect, listened to them carefully, and provided clear explanations to them. 90.3% were satisfied with the hospital sanitary measures. 64.1% stated that they got written instructions at the time of discharge.

**Conclusion:**

The satisfaction level of patients was high for all the studied domains, and there were a small number of complications with overall good surgical outcomes. That indicates that all the actions and policies that were implemented during the pandemic were proven beneficial for the patients. It is recommended to continue those measures until the COVID-19 pandemic is over.

## Highlights

• The COVID-19 pandemic has greatly affected health care systems across developing and developed countries.
• This study was to evaluate the impact of the COVID-19 pandemic on patient satisfaction and surgical outcomes.
• Overall, the rate of adverse surgical outcomes was low and we were able to achieve sustained levels of positive patients' satisfaction rates.
• We believe it may be safe to perform selective surgical procedures after taking stringent precautionary measures.

## 1 Introduction

In December 2019, mainland China witnessed a suspicious cluster of diseased patients manifesting as Typical Pneumonia, which was later corroborated as yet another Coronavirus outbreak after almost two decades of Severe Acute Respiratory Syndrome Coronavirus (SARS-CoV) from 2002 to 2003 [1]. The World Health Organization declared COVID-19 as Public Health Emergency International Concern on January 30, 2020, [2]. The current number of confirmed cases (20,424,156) and deaths (742,443) as of August 11, 2020 portrays a dismal scenario, not to mention how the COVID-19 pandemic has emaciated the globe physically, emotionally, and financially. Over 200 countries have been stricken thus far and Saudi Arabia presently stands at 291,468 confirmed cases and 3,233 deaths as of August 11, 2020 [3].

Unprecedented circumstances like recent COVID-19 pandemic put immense pressure on healthcare service providers to reshape the hospital infrastructure and policies to deter the spread of deadly infections and ensure smooth functioning of healthcare delivery [4]. One such healthcare domain that requires thoughtful guidelines revision is surgical care services. There must be a pandemic-preparedness plan that could assist in maintaining a fine balance between surgical care services delivery and minimal risk of nosocomial COVID-19 infection while conducting both elective and emergency surgeries. While the primary aim of the revised policies is to shield patients as well as healthcare personnel from preventable infections, patients may not fully understand the essence of imposed rules and regulations.

Patient-centered care is a prerequisite of high-quality healthcare delivery and the opposite is true for poor patient satisfaction which may affect healthcare outcomes [5]. One study by Lobo Prabhu et al. (2018) documented a significant relationship between the satisfaction of patients and postoperative surgical complications and 30-day readmission [6]. Patient satisfaction has been reported to influence the communication, compliance, promptness to seek medical consultation, continuity of care, and patients’ understanding and retention of the given information, all of which are indispensable for the high quality delivery of clinical care [[7], [8], [9]].

In light of the above, systematized research is needed to understand the change, if any, in the dynamics of patient care, satisfaction, and post-surgical outcomes with regards to revised infrastructure and policies in hospitals in the wake of infectious pandemics like COVID-19. Therefore, the current study was conducted.

## 2 Material and methods

### 2.1 Study setting, design, and procedures

A retrospective cross-sectional study was conducted from 13-3–2020 to 26-4-2020 to investigate the impact of COVID-19 pandemic on patient satisfaction and surgical outcomes at King Khalid University Hospital in Saudi Arabia, tertiary care hospital, after the ministry of health declared COVID-19 pandemic in the Saudi Arabia on 13-3-2020.

Study participants were all patients who came to the hospital to seek surgical care for various medical problems. Patient demographics and clinical characteristics were collected for all procedures done after the covid-19 pandemic announcement in Riyadh, Saudi Arabia on March 13, 2020. All patients were then interviewed over the phone after obtaining verbal consent to assess their satisfaction by a single trained data collector to minimize variation in the way the questions were presented to different patients. This work has been reported in line with the STROCSS [10].

### 2.2 Questionnaire

Hospital Consumer Assessment of Healthcare Providers and Systems (HCAHPS) questionare was used which is a standard and validated structured satisfaction questionnaire used in many published studies [[11], [12], [13], [14]].

The HCAHPS questionnaire included one dimension on overall satisfaction with hospital care, as well as items concerning patients' recommendation of their hospital's recommendation to someone else. Patients were also given a chance to self-rate their overall health with a specific focus on mental and physical health. Patients' satisfaction with hospital care and their evaluation of their hospital's reputation was measured using ordinal responses such as always, usually, sometimes, never or strongly agree, agree, disagree and strongly agree or worst, poor, fair, good, very good and excellent for some items [11].

#### 2.2.1 Changes in policies and precautionary measures in the hospital following COVID-19 pandemic declaration

Following the declaration of COVID-19 pandemic in Saudi Arabia, a dedicated hospital committee was established, which consisted of a member of each surgical specialty and supervised by the chairman of the surgery department to approve any submitted cases for surgery. In addition to emergency operations, only elective oncology and selected non-oncology operations were allowed to be performed. Additional steps and precautionary measures were taken to minimize exposure to patients such as preventing visitors from entering the hospital and decreasing team members in each health care discipline to create a rotationary system. Moreover, all the hospital services minimized their duties to provide a back up team in case of individuals suspected of contracting COVID-19 were isolated for 14 days or until the results of the two consecutive PCR swabs resulted negative. Furthermore, All surgical wards were merged to arrange separate wards for covid19 patients only. The hospital management closed most of the hospital gates and all clinics were closed except for selected urgent visits, otherwise, virtual clinics were conducted over the phone. All individuals in the hospital vicinity were prohibited from walking into the hospital areas and the majority of the coffee shops and mosques were closed with restrictions applied to large gatherings. Moreover, overall hospital capacity was decreased as patients were kept in single rooms rather than the two-patient per room arrangement previously utilized. Medical and surgical intensive care units (ICUs) were merged to establish a COVID19 ICU area and as a result, overall bed capacity in ICUs was also decreased. An admission pathway was initiated for identification and management of COVID-19 cases which start from the triage area at the emergency department or admission office. In case of respiratory illness screening tool (RIST) score equal or above of 4, the patient must wear mask and directed for further assessment and COVID-19 swab.

#### 2.2.2 Changes in the operating area and room following COVID-19 pandemic declaration

A Special area was designated to receive the high-risk patients to avoid unnecessary waiting in the holding area. In addition, it was mandatory for the anesthesia consultants to intubate all patients in a separated negative pressure room after using personal protection equipment (PPEs) followed by shifting the patient to another room for the surgery. All stand-by surgical equipments were kept outside the operating room and were covered properly to protect them from being contaminated and only brought inside the room if needed. It was decided to decrease the number of the surgical team members to 2–3 and limit that to the most senior trainees and physician assistants. In addition, all laparoscopic surgeries were considered as aerosol procedures, therefore, full protection measures were taken and patients were kept in the same room for recovery. It was also ensured to discharge the patients promptly once they met all discharge criteria.

### 2.3 Statistical analysis

Descriptive analyses was used to report frequencies and proportions to describe the characteristics of the study population for the categorical variables such as gender, language, education, race, type of surgery, and surgical outcomes. The normality assumption was checked for continuous variables by histograms superimposed with the normal curve and all continuous variables were skewed, therefore, median and interquartile range (IQR) were used for the continuous variables such as the age of the patients and length of stay (days) in the hospital and frequencies and proportions for the categorical variables. The proportion for all the satisfaction questionnaires were calculated and reported the numbers and proportions for ordinal responses for satisfaction items or questions. All analyses were performed using SPSS version 25.

## 3 Results

1. Demographic and Clinical Characteristics of the patients who underwent surgery during COVID-19 in KKUH

**Fig. 1:**
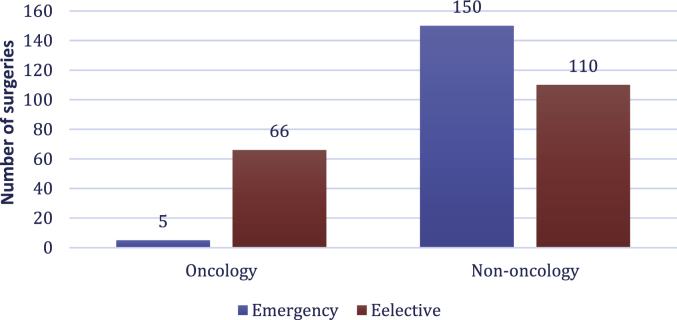
Distribution of cases operated during the study period in KKUH (n = 331).

Table 1 describes the demographic and clinical characteristics of the patients who underwent surgery at KKUH. The median age of the patients was 53 years (IQR of 33) with three-fourths of the patients being females. 78.9% (n = 176) of the patients had an education higher than the 8th grade and 98% (n = 219) of the patients reported speaking the Arabic language at their homes. Based on RIST score, screening test for COVID-19 was performed on 20.2% (n = 67) of patients, none of which was found to be positive. 260 cases (78.6%) were non-oncology while 71 (21.1%) were for oncology cases. Of the total surgeries performed in the non-oncology cases, 42.3% (n = 110) were elective, and 57.7% (n = 150) were emergency surgeries. On the other hand, of the total 71 surgeries performed in the oncology cases, 7% (n = 5) were emergency procedures and 93% (n = 66) were elective procedures as shown in Fig. 1.

**Table 1:** Demographic and Clinical Characteristics of the patients underwent surgery during COVID-19 at King Khalid University Hospital (n = 331).

	*Median/n*	*IQR/%*
**Patient's Age (Years)**	53	33
**Education**^a^		
8th grade or less	47	21%
Some high School but not graduate	14	6%
High School Graduate	52	23%
Some college or 2 year degree	11	5%
4 year college graduate	45	20%
More than 4 year college degree	54	24%
**Language**^a^		
Arabic	219	98%
Others	4	2%
**Race**^a^		
Arab	218	98%
Asian/American Indian/African American	5	2%
**Gender**		
Male	99	30%
Female	232	70%
**Type of Surgery**		
Elective	176	53%
Emergency	155	47%
**Type of Cases**		
Oncology	71	21.4%
Non-Oncology	260	78.6%

a The n for these variables is 223 as these questions were only asked from those who responded to the series of satisfaction questions.

**Fig. 2:**
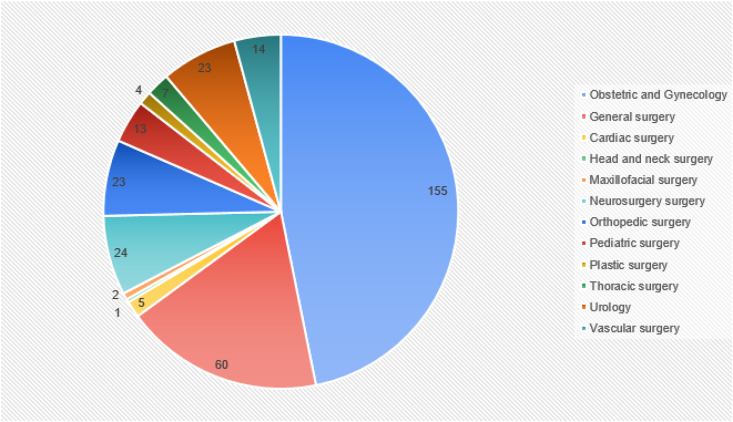
Distribution of cases by the specialty in KKUH (n = 331).

Regarding the distribution of the cases by different specialties, we found that 155 cases were managed by obstetric and gynecology, followed by 60 cases done by general surgery, then 24 and 23 cases done by neurosurgery and urology, respectively as shown in Fig. 2.

2. Surgical outcomes of the patients underwent surgery during COVID-19 pandemic

Table 2 summaraizes the main surgical outcomes. The average length of stay was 3 days with interquartile range (IQR) of 5 days. 12.4% (n = 41) of the patients required admission to the ICU and 0.9% (n = 3) were admitted to the HDU, all were due to either observation or routine post-operative care. The median length of stay in the ICU was 1 day with the IQR of 2 days. A 1.8% of patients (n = 6) were re-admitted within 30 days to the hospital. Surgical complication were observed in 10.9% (n = 36)of patients. Most of the complications were infectious, mainly surgical site infections followed by non-infectious like bleeding and hoarsness as shown in Table 2.

**Table 2:** Descriptives for the Surgical outcomes for the patients underwent surgical procedures at KKUH (n = 331).

	Median/n	IQR/%
**Length of Stay in the Hospital (days)**	3	5
**Admission in ICU**	41	12%
**Length of stay in ICU (days)**	1	2
**Admission in HDU**	3	0.9%
**Complications**	36	11%
**Type of Complications**		
Any infection	14	39%
Infected prosthesis and collection	3	8%
Bleeding and Hematoma	5	14%
Hoarseness	3	8%
Pulmonary	2	6%
Adrenal and Kindney related	2	6%
Neurology complications	3	8%
Urology Complications	4	11%
**Mortality**	3	0.9%
**Readmission in one month**	6	2%

Overall there were three mortality cases (0.9%), all of which were emergency non-oncology cases. Of these three deaths, one case died intra-operatively due to severe thoracoabdominal trauma. The other two cases died in the ICU after neurosurgical intervention for sever brain injury and intra-cranial hemorrage.

3. Patients' satisfaction rating for attending nurses and doctors' behaviors and for the environment of the hospital during hospital stay

Table 3 illustrates patients' satisfaction ratings for attending nurses' and doctors’ behaviors as well as for the environment of the hospital during admission. With respect to the care administered, 77.6% and 93% of the patients reported that nurses and doctors, respectively, always treated them with courtesy and respect, listened to them carefully, and provided comprehensible answers to all their questions. Moreover, 91% of the patients reported that they always needed help from the nurses or other hospital staff in getting to the bathroom or in using a bedpan, while 76.8% reported that the hospital staff always took the time to explain which medicine was being administered and for what purpose.

**Table 3:** Patients' satisfaction ratings for attending Nurses and doctor's ≥ behaviors and for the environment of the hospital during hospital stay at King Khalid University Hospital (n = 223).

*Rating by patient*										
	*Nurses Behavior (n(%)*			*Hospital Staff's Help(n(%)*		*Doctor's Behavior(n(%)*			*Environment of the Hospital (n(%)*	
	Nurses treatment with courtesy and respect	Nurses listened carefully	Nurses explained things in understandable manner	Need help from nurses or other hospital staff in getting to the bathroom or in using a bedpan	How often did hospital staff tell you what the medicine was for	Doctor's treatment with courtesy and respect	Doctors listened carefully	Doctors explained things in understandable manner	Room and bathroom were kept clean	Area around patients room quiet at night
**Always**	173 (77.6)	170 (76.2)	172 (77.1)	86 (91)	106 (76.8)	209 (93.7)	209 (93.7)	209 (93.7)	199 (89.2)	179 (91.3)
**Usually**	14 (6.3)	15 (6.7)	19 (8.5)	16 (17)	4 (2.8)	2 (0.9)	3 (1.3)	2 (0.9)	9 (4.0)	4 (2.0)
**Sometimes**	24 (10.8)	26 (11.7)	24 (10.8)	12 (12.7)	10 (7.2)	9 (4.0)	9 (4.0)	8 (3.6)	11 (4.9)	9 (4.5)
**Never**	11 (4.9)	12 (5.4)	8 (3.6)	15 (16)	18 (13.0)	3 (1.3)	2 (0.9)	4 (1.8)	4 (1.8)	4 (2.0)

***Rating of the patients at the time of discharge* (n** = **223)**			
***Rating by patient***	**Staff took me and my caregivers preferences into account*(n(%)***	**Patient understood the purpose for taking medicines*(n(%)***	**Patient had a good understanding to managing their health*(n(%)***
**Strongly Agree**	38 (17.0)	**54(24.2)**	56 (25.1)
**Agree**	147 (65.9)	141 (63.2)	137 (61.4)
**Disagree**	33 (14.8)	23 (10.3)	24 (10.8)
**Strongly Disagree**	5 (2.0)	5 (2.0)	6 (3.0)

When asking the patients about the environment of the hospital, 89.2% of the patients reported that housekeeping ensured that their room and bathroom were kept clean all the time. Similarly, 91.3% of the patients also reported that the area around their premises or surrounding their room was quiet at night without any disturbance. When asking the patients about whether staff considered their and their caregiver's preferences into account, 85% of the patients agreed (strongly agree or agree). Similarly, 85% of the patients agreed (strongly agree or agree) that they understood the purpose of taking their prescribed medication and a similar proportion of the patients (86.5%) agreed (strongly agree or agree) that they had a good understanding of how to manage their health.

4. Patients' opinion about the overall rating of the hospital and their health at the time of discharge

Table 4 demonstrates patients’ opinions about the overall rating of the hospital and their health at the time of discharge. More specifically, it shows that 88% of the patients were discharged directly to their homes and only 1.3% went to another health care facility. Around two-thirds of the patients (67.2%) stated that hospital staff ensured that all their concerns were addressed at the time of discharge. A similar proportion (64.1%) stated that they got written instructions at the time of discharge.

**Table 4:** Patients’ opinion about the overall rating of the hospital and their health at the time of discharge at King Khalid University Hospital (n = 223).

Variable	*Response*	*n(%)*
**Patients' destination after discharge**	Own home	197 (88)
	Another health facility	3 (1.3)
	Someone else's home	23 (10.3)
**Hospital staff talked with patient about any kind of help needed by them at the time of discharge**	Yes	150 (67.2)
	No	73 (32.7)
**Patient got information in writing about symptoms or health problems at the time of discharge**	Yes	143 (64.1)
	No	80 (35.8)
**Rating of the hospital during stay**	Worst	1 (0.4)
	Poor	3 (1.3)
	Fair	5 (2.2)
	Good	28 (12.5)
	Very Good	89 (39.9)
	Excellent	97 (43.5)
**Recommending this hospital to friends and family**	Yes	209 (93.7)
	No	14 (6.2)
**Self-rating overall health**	Worst	0 (0)
	Poor	1 (0.4)
	Fair	8 (3.6)
	Good	15 (6.7)
	Very Good	14 (6.3)
	Excellent	184 (82.5)
**Self-rating of mental or emotional health**	Worst	0 (0)
	Poor	4 (1.8)
	Fair	7 (3.1)
	Good	12 (5.4)
	Very Good	13 (5.8)
	Excellent	187 (84)

Regarding the rating of the hospital during admission, 85% of the patients stated that their stay was good or excellent. 94% reported that they would recommend the hospital to their friends and family members when seeking medical care. When rating their overall health, 82.5% of the patients self-rated their overall health as excellent, and 6.3% reported it as very good. Similarly, 84% reported their mental and emotional health as excellent and 5.8% reported it as very good.

## 4 Discussion

Major precautionary measures were applied to the health care system during the COVID-19 pandemic. This study was conducted to assess the surgical outcomes and patient's satisfaction during this global crisis. Overall, study findings revealed that 20% of the patients underwent COVID-19 test after applying RIST score, and all were negative. The data also demonstrated that half of the patients underwent elective surgery and the other half underwent emergency surgical procedures with low overall complications, mortality, and readmissions.

During the outbreak in Spain, Gallego MÁ et al. reported that the confirmed COVID-19 infection rate was 7% and 11.1% in elective and emergency cases, respectively. The mortality rate was found to be 1.6%, all due to respiratory failure [15]. Similarly, COVID-19 was confirmed in 7% of the patients in the study by Martino MD et al., with a mortality rate of 1.4% due to severe respiratory symptoms [16]. In comparison to our result, none of the patients were confirmed to have COVID-19, and the mortality rate was 0.9%, all in emergency cases due to severe brain injury and major thoracoabdominal trauma.

In contrast, COVIDSurg Collaborative group conducted an international, multicentre, cohort study with a total of 1128 confimred SARS-Cov patients to evaluate the perioperative pulmonary complication and mortality. Pulmonary complications were found in 51.2%, and the overall mortality rate was 23.8% [17]. In another study conducted in Wuhan, China, it was noticed that 44.1% of the positive COVID-19 patients who underwent elective surgery deteriorated and were admitted to the ICU with a mortality rate of 20.5% [18]. The reasons for the high complications and mortality rate in these studies compared to our study could be that only positive COVID-19 cases were included and a country such as China was the epicenter of COVID-19.

During study period, there were confirmed COVID-19 cases among health care workers (5 cases) and patients (11 cases) in our hospital, and the hospital administration implemented new polices and strict preventative measures to minimize the rate of adverse surgical outcomes among patients, which is also confirmed by the higher level of patient's satisfaction as detailed above.

Concerning patients' satisfaction, we found that overall, most of our patients were satisfied with the staff behavior and the services of the hospital. The majority of them were happy with nurses' and doctor's behavior and communication during their hospital stay. With respect to the overall rating of the hospital and recommending the same hospital to others, most of the patients reported that the hospital is excellent and they would recommend this to others.

To our knowledge, no other study conducted so far assessed patients' satisfaction in a similar context, we could compare our study findings favourably with other studies in the literature, which have been conducted before COVID-19 era [19]. This is also justifiable because none of the study participants was found to be positive for COVID-19 in this analysis, which further makes these patients similar to the pre-COVID-19 era despite the rigorous measures undertaken in our hospital during the pandemic and the difficulties our hospital was facing. These findings can be explained by the fact that nursing care is the main supportive provision to the admitted patients, and nursing staff also comprise the principal percentage of the health sector mainly for the in-patient service [20,21]. Our findings regarding the patient's satisfaction are higher than other studies conducted in different settings where the proportion of satisfied patients was less than 60% [22,23].

It is also important to highlight that in the past, authors conducted a satisfaction survey study in our center (KKUH) and they found that overall patients were not satisfied with the communication of doctors [24]. Another study was conducted in the same center (KKUH), which aimed to assess the satisfaction of nursing services and this study found that patients were highly satisfied with the skills of the nurses but not with the communication [25]. These findings also contradict with our study findings in terms of satisfaction domains such as communication. Overall, our study demonstrated a higher level of patient satisfaction when compared to the other studies being carried out in the past. These differences in the patients’ satisfaction could be due to several reasons. First of all, this could be becausethe amount of care the patient received during this COVID-19 pandemic have been beyond their expectations during these difficult times. In addition, KKUH implemented many policies with stringent control measures within and outside the operation room as highlighted. The higher level of satisfaction in our study indicates that these measures introduced by the hospital worked successfully to a larger extent during this COVID-19 pandemic. The overall satisfaction of the patients could also be due to the fact that none of them was positive for COVID-19, which provided a major relief to the patients and thus augmented their satisfaction. The differences between our study and other international studies could be due to the time differences as well as some important parameters such as sample size, type of questionnaire, and modality of administration. Lastly, the level of satisfaction is a subjective phenomenon that could vary easily from one patient to the next and from one hospital to the other or even over a period of time. Conducting a patient satisfaction survey could provide a pathway to the hospital authorities to improve their services and meet the demands of the patients by providing the quality of care in a timely manner.

### 4.1 Strengths and limitations

Although multiple studies have been conducted in the past to assess patient satisfaction in a number of ways by using a range of different questionnaires, our study explored patient satisfaction after implementing different policies and precautionary measures during the era of COVID-19 using a tested and validated questionnaire. So far, to the best of our knowledge, no other study have conducted a patients’ satisfaction and surgical outcomes assessment simultaneously during the COVID-19 pandemic.

Some potential caveats render the study findings subject to cautious interpretaion. First, the retrospective cross-sectional nature of the survey does not allow temporality to be unambiguously determined. Secondly, we did not collect data on the important variables such as the socio-economic status of the patient and previous surgery or hospital admission.

## 5 Conclusion

Overall the rate of adverse surgical outcomes was low in our study and we were able to achieve sustained levels of positive patient experience and satisfaction rates in one of the largest institutions in Saudi Arabia.

We believe it may be safe to perform selective surgical procedures during this pandemic after taking stringent precautionary measures.

## Ethical approval

Ethical approval was give by IRB in King Saud University, reference No. E-20-4954.

## Sources of funding

None.

## Author contribution

Thamer A Bin Traiki: study design and final review.

Sulaiman A AlShammari: Writing and data analysis.

Mohammed N AlAli: Writing and data analysis.

Nadia A Aljomah: Data collection and writing.

Noura S Alhassan: Study design and result review.

Khayal A Alkhayal: Writing and final review.

Omar A Al-Obeed: Data analysis and final review.

Ahmad M Zubaidi: Study design and discussion review.

## Research registration unique identifying number (UIN)

- Registry used: http://www.researchregistry.com
- Unique Identifying number: researchregistry5761
- Registration ID: 5761
- Hyperlink to your specific registration:

https://www.researchregistry.com/register-now#home/registrationdetails/5ef8ad990905910018f3bdbb

## Guarantor

Thamer A Bin Traiki, Sulaiman AlShammari, and Mohammed N AlAli,; all are taking the full responsibility for submitted work.

## Provenance and peer review

Not commissioned, editor reviewed.

## Declaration of competing interest

All authors have no conflicts of interest or financial ties to disclose.
